# Crimean-Congo hemorrhagic fever virus strains Hoti and Afghanistan cause viremia and mild clinical disease in cynomolgus monkeys

**DOI:** 10.1371/journal.pntd.0008637

**Published:** 2020-08-13

**Authors:** Robert W. Cross, Abhishek N. Prasad, Viktoriya Borisevich, Joan B. Geisbert, Krystle N. Agans, Daniel J. Deer, Karla A. Fenton, Thomas W. Geisbert

**Affiliations:** 1 Galveston National Laboratory, University of Texas Medical Branch, Galveston, Texas, United States of America; 2 Department of Microbiology & Immunology, University of Texas Medical Branch, Galveston, Texas, United States of America; Saudi Ministry of Health, SAUDI ARABIA

## Abstract

**Background:**

Development of vaccines and therapies against Crimean-Congo hemorrhagic fever virus (CCHFV) have been hindered by the lack of immunocompetent animal models. Recently, a lethal nonhuman primate model based on the CCHFV Hoti strain was reported. CCHFV Hoti caused severe disease in cynomolgus monkeys with 75% lethality when given by the intravenous (i.v.) route.

**Methodology/Principal findings:**

In a series of experiments, eleven cynomologus monkeys were exposed i.v. to CCHFV Hoti and four macaques were exposed i.v. to CCHFV Afghanistan. Despite transient viremia and changes in clinical pathology such as leukopenia and thrombocytopenia developing in all 15 animals, all macaques survived to the study endpoint without developing severe disease.

**Conclusions/Significance:**

We were unable to attribute differences in the results of our study versus the previous report to differences in the CCHFV Hoti stock, challenge dose, origin, or age of the macaques. The observed differences are most likely the result of the outbred nature of macaques and low animal numbers often used by necessity and for ethical considerations in BSL-4 studies. Nonetheless, while we were unable to achieve severe disease or lethality, the CCHFV Hoti and Afghanistan macaque models are useful for screening medical countermeasures using biomarkers including viremia and clinical pathology to assess efficacy.

## Author summary

Crimean-Congo hemorrhagic fever virus (CCHFV) is a tick-borne virus in the family *Nairoviridae*, and is the etiological agent of a severe and potentially fatal hemorrhagic fever disease in humans (CCHF). Despite being the most geographically widespread tick-borne arbovirus, CCHFV remains understudied, and the lack of specific pre- and post-exposure medical countermeasures for CCHFV infection has led to its inclusion in the World Health Organization (WHO) list of pathogens requiring urgent research attention. Development and approval of vaccines and therapeutics against CCHFV necessitates efficacy testing in animal models that accurately recapitulate human disease. Recently, a lethal model for CCHF in cynomolgus macaques utilizing the Hoti strain of CCHFV was reported. We sought to verify and expand on this model by inclusion of an additional CCHFV strain (Afghanistan), animals from different geographic origins, and assessing the impact of age. In contrast to the previous work, none of the animals in our study developed severe or lethal disease. Importantly, we were unable to attribute these contrasting findings with differences in experimental parameters, implying that the discrepancy between the findings in the previous study and ours is likely due to the outbred nature of the animals and the small number of animals used in BSL-4 studies.

## Introduction

Crimean-Congo hemorrhagic fever virus (CCHFV) is a member of the genus *Orthonairovirus* (family *Nairoviridae*) and causes significant disease, CCHF, in humans [1, 2]. CCHFV has a broad geographic range and is endemic in more than 30 countries in Eurasia, Africa, and the Middle East. As a result of high case fatality rates, the ability to spread easily by human-to-human contact, and the potential for aerosol release, CCHFV, like most hemorrhagic fever (HF) viruses, is classified by several US Government agencies as a Category A priority pathogen. The World Health Organization (WHO) has also recently placed CCHFV on their list of top 10 priorities for emerging virus research [3]. There are currently no FDA-approved CCHF vaccines or antiviral drugs for human use; ribavirin is indicated in some protocols, although controversy exists regarding its efficacy in treating CCHF [4]. CCHFV is usually transmitted to humans by bites from ixodid ticks of the genus *Hyalomma*, or through contact with blood or tissues of viremic livestock or patients [5]. Interestingly, while both wild and domestic animals develop viremia, humans and potentially nonhuman primates (NHPs) appear to be the only species where CCHFV infection causes overt disease. Human infection by CCHFV is characterized by headache, fever, myalgia, and petechial rash. In severe cases, circulatory shock and disseminated intravascular coagulation may occur with necrotic hepatitis. The global case fatality rate for CCHF has averaged around 30%; however, it varies geographically from a low of approximately 5% in Turkey [6] to a high of nearly 60% in the United Arab Emirates [7].

Currently, there are no vaccines for CCHF available for human use. Development of vaccines has been hindered by the requirement to study CCHFV in high containment Biosafety Level (BSL)-4 facilities as well as the lack of available animal models. While the STAT-1 and IFNAR knockout mouse models are a significant advance in terms of animal models for CCHF [8–10], there remains a clear need for immunocompetent animal models that faithfully reproduce human CCHF. Numerous attempts to develop such models have been made [11]. CCHFV-infection studies of mice, rats, guinea pigs, hamsters, rabbits, sheep, calves, and donkeys have demonstrated that these species exhibit low to undetectable viremia and clear the infection without overt signs of illness [12, 13]. Historically, a very small number of studies have assessed the pathogenic potential of CCHFV in NHPs, with none showing any promise regarding the development of a NHP model [12, 14, 15]. However, a lethal NHP model of CCHF using cynomolgus monkeys was recently reported [16]. In brief, this model utilizes the Hoti strain of CCHFV. Importantly, Haddock et al. reported that intravenous (i.v.) administration of 1x10^5^ median tissue culture infectious dose (TCID_50_) of CCHFV Hoti resulted in 75% lethality in macaques and that the disease observed faithfully reproduced human clinical manifestations. Here, we attempted to repeat these findings as well as to assess the pathogenic potential of a different strain of CCHF from Afghanistan in cynomolgus monkeys.

## Methods

### Virus isolates

A CCHFV Afghanistan strain passage 4 (P4) isolate was obtained from the European Virus Archive (https://www.european-virus-archive.com). This virus was passed one time in SW-13 cells (ATCC CCL-105) to produce a P5 stock. Three different CCHFV Hoti stocks were obtained or prepared for this project. CCHFV Hoti (P7) was obtained from the European Virus Archive (https://www.european-virus-archive.com). In addition, the same CCHF Hoti P9 stock used in the previous study [16] that observed 75% lethality in macaques was kindly provided by Dr. Heinz Feldmann (NIAID/NIH, Rocky Mountain Laboratories, Hamilton, MT). This stock was passed one time in SW-13 cells (ATCC CCL-105) to produce a P10 stock.

### Animal challenge

Prior to challenge with CCHFV, all animals were anesthetized by intramuscular injection of ketamine. In the first study, two Chinese origin cynomolgus monkeys (one male and one female, 4–5 years of age, 3–4 kg) were exposed i.v. with a target dose of 2x10^5^ pfu of cell culture passage 5 (P5) CCHFV Afghanistan while two Chinese origin cynomolgus monkeys (one male and one female, ~ 4–5 years of age, 3–4 kg) were exposed i.v. with a target dose of 2x10^5^ pfu of P10 CCHFV Hoti. In the second study, two Chinese origin cynomolgus monkeys (one male and one female, ~ 4 years of age, 3.3–3.7 kg) and two Mauritius origin cynomolgus monkeys (both males, ~ 4 years of age, 4.6–5.3 kg) were exposed i.v. with a target dose of 2x10^5^ pfu of P10 CCHFV Hoti while one Chinese origin cynomolgus monkey (male, ~ 4 years of age, 3.8 kg) and one Mauritius origin cynomolgus monkey (female, ~ 4 years of age, 3.9 kg) were exposed i.v. with a target dose of 2x10^5^ pfu of P9 CCHFV Hoti. In the third study, two Chinese origin cynomolgus monkeys (one male and one female, 3–8 years of age, 3–5.6 kg) were exposed i.v. with a target dose of 2x10^5^ pfu of P7 CCHFV Hoti. In the fourth and final study, two Mauritius origin cynomolgus monkeys (both males, >18 years of age, 8.1–9.0 kg) were exposed i.v. with a target dose of 2x10^5^ pfu of P5 CCHFV Afghanistan while one Mauritius origin cynomolgus monkey (male, >18 years of age, 9.6 kg) was exposed i.v. with a targer dose of 2x10^5^ pfu of P10 CCHFV Hoti. At various times before and after virus challenge, animals from all four studies were monitored for clinical signs of illness including temperature, respiration quality, and clinical pathology.

### Ethics approval

The animal studies were performed at the Galveston National Laboratory, University of Texas Medical Branch at Galveston (UTMB) and were approved by the UTMB Institutional Animal Care and Use Committee. This facility is fully accredited by the Association for Assessment and Accreditation of Laboratory Animal Care International and has an approved OLAW Assurance # D16-00202 (A3314-01). All steps were taken to ameliorate the welfare and to avoid the suffering of the animals in accordance with the ‘‘Weatherall report for the use of nonhuman primates” recommendations. Animals were housed in adjoining individual primate cages allowing social interactions, under controlled conditions of humidity, temperature, and light (12-hour light/12-hour dark cycles). Food and water were available ad libitum. Animals were monitored (pre- and postinfection) and fed commercial monkey chow, treats and fruit twice daily by trained personnel. Environmental enrichment consisted of commercial toys. All procedures were conducted by trained personnel under the oversight of an attending veterinarian and all invasive clinical procedures were performed while animals were anesthetized with Ketamine or Telazol. All animals were monitored daily and scored for disease progression with an internal CCHF scoring protocol approved by the UTMB IACUC. The scoring changes measured from baseline included posture/activity level, attitude/behavior, food and water intake, weight, respiration and disease manifestations such as visible rash, hemorrhage, ecchymosis or flushed skin. A score of ≥9 indicated that an animal met the criteria for euthanasia. Animals were euthanized at the study endpoint using a sodium pentobarbital-based commercial euthanasia solution.

### RNA isolation from CCHFV-infected macaques

On procedure days, 100 μl of blood from K2-EDTA collection tubes was collected prior to centrifugation, and was added to 600 μl of AVL viral lysis buffer with 6 μL carrier RNA (Qiagen) for RNA extraction. For tissues, approximately 100 mg was stored in 1 ml RNAlater (Qiagen) for at least 4 days for stabilization. RNAlater was completely removed, and tissues were homogenized in 600 μl RLT buffer and 1% Betamercaptoethanol (Qiagen) in a 2 mL cryovial using a tissue lyser (Qiagen) and 0.2mm ceramic beads. The tissues sampled included axillary and inguinal lymph nodes, liver, spleen, kidney, adrenal gland, lung, pancreas, urinary bladder, ovary or testis, and eye. All blood samples were inactivated in AVL viral lysis buffer, and tissue samples were homogenized and inactivated in RLT buffer prior to removal from the BSL-4 laboratory. Subsequently, RNA was isolated from blood using the QIAamp viral RNA kit (Qiagen), and from tissues using the RNeasy minikit (Qiagen) according to the manufacturer’s instructions supplied with each kit.

### Detection of CCHFV load

RNA was isolated from whole blood using the Qiamp viral RNA kit (Qiagen) using 100 μL of blood into 600 μL of viral lysis buffer AVL. For tissues, approximately 100 mg was stored in 1mL RNALater (Qiagen) for at least 4 days at 4°C for stabilization. Tissues were removed from RNALater and homogenized in 600 μL RLT buffer and 1% β-mercaptoethanol (Qiagen) using a tissue lyser and 0.2mm ceramic beads. RNA was then isolated from tissue homogenates using the RNeasy minikit (Qiagen). Viral load was assessed using primers/probe targeting the nucleocapsid protein (N) on the S segment of CCHFV gene through real-time quantitative PCR (RT–qPCR), with the probes used being strain specific. The CCHFV-Afghanistan probe was 5'6- carboxyfluorescein (6FAM)-ATC TAC ATG CAC CCT GCT GTG C-3'-Black Hole Quencher 1 (3BHQ_1) (Integrated DNA Technologies, IDT) [17] and the CCHFV-Hoti probe was 5’(6FAM)-AGA AGG GGC TTG AGT GGT T-3’-Dabcyl Quencher 3 (Dab) (IDT) [18] CCHFV RNA was detected using the CFX96 detection system (BioRad Laboratories) in one-step probe RT–qPCR kits (Qiagen) with the following cycle conditions: 50°C for 10 min, 95°C for 10 s, and 45 cycles of 95°C for 10 s and 54°C for 30 s. Threshold cycle (CT) values representing CCHFV S genomes were analyzed with CFX Manager Software, and data are shown as genome equivalents (GEq). To create the GEq standard, RNA from strain specific CCHFV stocks was extracted. The number of CCHFV S genomes was calculated using Avogadro’s number and the molecular weight of the CCHFV genome. Limit of detection was 1 × 10^3^ GEq/ml for CCHFV-Afghanistan and 1 × 10^4^ GEq/ml for CCHFV-Hoti.

Virus titration was performed by plaque assay using SW-13 cells (ATCC CCL-105) from all plasma and tissue samples as previously described [19]. Briefly, increasing 10-fold dilutions of the samples were adsorbed to SW-13 cell monolayers in duplicate wells (200 μl) and overlaid with 1.25% Avicel RC-581 (FMC BioPolymer, Philadelphia, PA) in 1x Eagles minimum essentials medium (MEM) with 5% FBS and 1% P/S). After 3–4 days, the overlay was removed and the monolayer stained with a 1X crystal violet/10% buffered formalin solution. The limit of detection was 25 PFU/ml for plasma.

### Serum neutralization assay

A plaque reduction neutralization test was employed to determine neutralizing antibody titers in serum. Sera was heat inactivated at 56°C for 30 minutes and then serially diluted two-fold starting at a 1:10 dilution and each dilution were incubated with ~ 100 PFU of the relevant CCHFV strain (Afghanistan or Hoti) for 1 h at 37°C. The virus:serum mixtures were then added to individual wells of 6-well plates of confluent SW-13 cell (ATCC CCL-105) monolayers in duplicate. After a one-hour incubation, cells were overlaid with 0.8% agarose in 2x Modified Eagle Medium (EMEM, LifeTechnologies). Plates were stained with neutral red 2 days after infection, and plaques were counted 24 h after staining. Endpoint titers were determined by assessing the serum dilution at which there was a ≥ 50% reduction in plaque counts compared to control wells.

### Hematology and serum biochemistry

Total white blood cell counts, white blood cell differentials, red blood cell counts, platelet counts, hematocrit values, total hemoglobin concentrations, mean cell volumes, mean corpuscular volumes, and mean corpuscular hemoglobin concentrations were analyzed from blood collected in tubes containing EDTA using a laser based hematologic analyzer (Beckman Coulter). Serum samples were tested for concentrations of albumin, amylase, alanine aminotransferase (ALT), aspartate aminotransferase (AST), alkaline phosphatase (ALP), blood urea nitrogen (BUN), calcium, creatinine (CRE), C-reactive protein (CRP), gamma-glutamyltransferase (GGT), glucose, total protein, and uric acid by using a Piccolo point-of-care analyzer and Biochemistry Panel Plus analyzer discs (Abaxis).

### Analysis of cytokines/chemokines

Circulating levels of 23 cytokines/chemokines in sera of CCHFV-infected macaques were measured using the Milliplex NHP cytokine magnetic bead panel plex (Millipore Sigma) following the manufacturer’s instructions. Analytes measured included IL-1β, IL-1 receptor antagonist (IL1ra), IL-2, IL-4, IL-5, IL-6, IL-8, IL-10, IL-12/23 (p40), IL-13, IL-15, IL-17, IL-18, IFN-γ, granulocyte colony-stimulating factor (G-CSF), granulocyte-macrophage colony-stimulating factor (GM-CSF), monocyte chemoattractant protein 1 (MCP-1), macrophage inflammatory protein 1α (MIP-1α), MIP-1β, tumor necrosis factor (TNF)-α, transforming growth factor-α (TGF-α), soluble CD40 ligand (sCD40L), and vascular endothelial growth factor (VEGF). Multianalyte profiling was performed using a Luminex-100 system; data were analyzed using Bio-Plex Manager software (Bio-Rad).

### Histopathology and immunohistochemistry

Necropsy was performed on all subjects, and tissue samples of all major organs were collected for histopathologic and immunohistochemical (IHC) examination. Tissue sections were deparaffinized and rehydrated through xylene and graded ethanol. Slides went through heat antigen retrieval in a steamer at 95°C for 20 mins in Sigma Citrate Buffer, pH6.0, 10× (Sigma Aldrich, St. Louis, MO). To block endogenous peroxidase activity, slides were treated with a 3% hydrogen peroxide and rinsed in distilled water. The tissue sections were processed for IHC using the Thermo Autostainer 360 (ThermoFisher, Kalamazoo, MI). Sequential 15 min incubations with avidin D and biotin solutions (Vector, Burlingame, CA) were performed to block endogenous biotin reactivity. Specific anti-CCHFV immunoreactivity was detected using a primary polyclonal rabbit-α-CCHFV-NP antibody (IBT BioServices, Rockville, MD) at a 1:3200 dilution for 60 mins. A secondary biotinylated goat-α-rabbit-IgG (Vector Laboratories, Burlingame, CA) at 1:200 dilution for 30 mins followed by Vector Horseradish Peroxidase Streptavidin, R.T.U (Vector) for 30 mins. Slides were developed with Dako DAB chromagen (Dako, Carpenteria, CA) for 5 mins and counterstained with Harris hematoxylin for 30 seconds. Tissue sections from historical control STAT-1 knock out mice infected with lethal doses of either Hoti or Afghanistan CCHFV were used as positive controls. Tissue sections from uninfected macaques were used as negative controls.

## Results

### NHP Experiment 1: Infection of cynomolgus monkeys with CCHFV Hoti and Afghanistan isolates results in mild clinical illness

**Fig 1:**
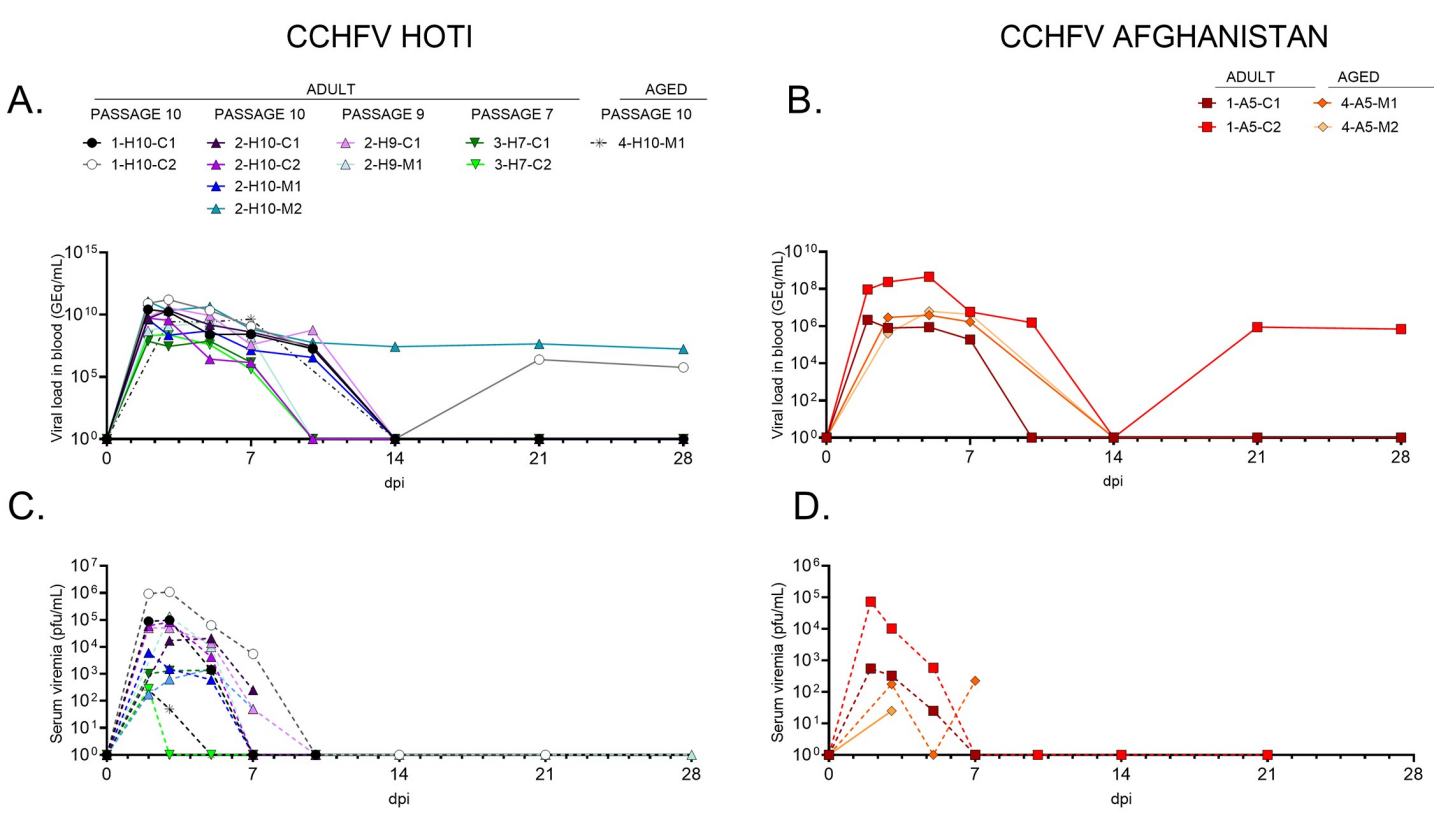
Circulating viral genomes and infectious particles in CCHFV infected macaques. Viremia was quantified RT-qPCR detection of viral genomes in whole blood (**A-B**), as well as by plaque titration of infectious viral particles in the plasma fraction (**C-D**). For all panels, data plotted is the mean of two technical replicates for each assay. Dashed lines on the y-axis of panels **A** and **B** indicates the limit of detection (LOD) for each assay.

We initially performed a small pilot study to confirm a previous report that CCHFV Hoti caused severe and usually lethal disease in cynomolgus monkeys when given by the i.v. route [16], and to assess the pathogenic potential of CCHFV Afghanistan in comparison. We chose CCHFV Afghanistan because it was also isolated from a fatal human case, was a low cell culture passage isolate that had not been passaged in mice, and was from a different CCHFV clade than CCHFV Hoti [20]. In this initial study, two Chinese origin cynomolgus monkeys (subjects 1-H10-C1 and 1-H10-C2) were exposed to 2x10^5^ pfu of P10 CCHFV Hoti by the i.v. route and two Chinese origin cynomolgus monkeys (subjects 1-A5-C1 and 1-A5-C2) were exposed to 2x10^5^pfu of P5 CCHFV Afghanistan by the i.v. route. All four macaques developed a transient viremia between days 2 and 5 after CCHFV challenge (**Fig 1**). However, while some transient changes in clinical pathology, such as leukopenia and thrombocytopenia, were observed in all four animals (**Tables 1 and 2**), none of the animals developed severe disease and all four animals survived to the study endpoint (day 28). Apart from subject 1-H10-C2, which exhibited minimal diffuse pallor of the liver, no significant findings were observed during necropsy of the animals in this study. Similarly, there was an absence of obvious histological lesions or CCHFV antigen labeling in all tissues analyzed, in contrast to tissues from murine historical controls which succumbed to disease from infection with CCHFV Afghanistan or CCHF Hoti (**S1A–S1P and S1Q–S1T Fig**). Neutralizing antibody titers against CCHFV were detected in all four macaques further confirming infection (**Table 3**).

**Table 1:** Clinical description of cynomolgus macaques following CCHFV Afghanistan challenge.

Subject No.	Sex	Origin	Age (years)	Virus passage	Sampling Days	Clinical illness	Clinical pathology
1-A5-C1	M	Chinese	~ 5	5	d0, 2, 3, 5, 7, 10, 14, 21, 28	None. Subject survived to study endpoint (d28).	Lymphopenia (d5, 21, 28); thrombocytopenia (d2, 3, 28); monocytopenia (d2, 5, 10–28); granulopenia (d3-28)
1-A5-C2	F	Chinese	~ 5	5	d0, 2, 3, 5, 7, 10, 14, 21, 28	Anorexia (d4). Subject survived to study endpoint (d28).	Thrombocytopenia (d2-7); monocytopenia (d2, 21, 28); granulopenia (d5, 7); lymphocytosis (d7-14); > 2-fold ↑ in AST (d3, 5); > 4-fold ↑ in CRP (d7)
4-A5-M1	M	Mauritius	~ 18	5	d0, 3, 5, 7, 14, 21	Anorexia (d2-4, 6, 9). Subject survived to study endpoint (d21).	Lymphopenia (d3, 7); monocytopenia (d3, 21); granulopenia (d3, 5); hypoglycemia (d21)
4-A5-M2	M	Mauritius	~ 18	5	d0, 3, 5, 7, 14, 21	Decreased appetite (d1-4, 6–10, 15). Subject survived to study endpoint (d21).	Lymphopenia (d3); thrombocytopenia (d7); monocytopenia (d21); granulopenia (d5, 7); lymphocytosis (d14)

Subject numbers are coded as [Experiment number]-[Virus and passage number]-[Animal origin and individual number]. Days after CCHFV Afghanistan challenge are in parentheses. Lymphopenia, granulopenia, monocytopenia, and thrombocytopenia are defined by a ≥35% drop in numbers of lymphocytes, granulocytes, monocytes, and platelets, respectively. Leukocytosis, lymphocytosis, monocytosis, and granulocytosis are defined by a two-fold or greater increase in numbers of white blood cells over baseline. Anorexia is defined as ≥25% decrease in baseline food intake. Fever is defined as a temperature more than 2.5°F over baseline, or at least 1.5°F over baseline and ≥ 103.5°F. Hypothermia is defined as a temperature ≤3.5°F below baseline. Hyperglycemia is defined as a two-fold or greater increase in levels of glucose. Hypoglycemia is defined by a ≥25% decrease in levels of glucose. Hypoalbuminemia is defined by a ≥25% decrease in levels of albumin. Hypoproteinemia is defined by a ≥25% decrease in levels of total protein. Hyperamylasemia is defined by a ≥2-fold increase in levels of serum amylase. Hypoamylasemia is defined by a ≥25% decrease in levels of serum amylase. Hypocalcemia is defined by a ≥25% decrease in levels of serum calcium. (ALT) alanine aminotransferase, (AST) aspartate aminotransferase, (ALP) alkaline phosphatase, (CRE) Creatinine, (CRP) C-reactive protein, (Hct) hematocrit, (Hgb) hemoglobin

**Table 2:** Clinical description of cynomolgus macaques following CCHFV Hoti challenge.

Subject No.	Sex	Origin	Age (years)	Virus passage	Sampling Days	Clinical illness	Clinical pathology
1-H10-C1	M	Chinese	~ 4	10	d0, 2, 3, 5, 7, 10, 14, 21, 28	None. Subject survived to study endpoint (d28).	Thrombocytopenia (d2-7); monocytopenia (d2, 28); granulopenia (d14); leukocytosis (d10); lymphocytosis (d10, 14); thrombocytosis (d14); granulocytosis (d3, 28); > 3-fold ↑ in ALT (d3-7); > 3-fold ↑ in AST (d3-7); > 3-fold ↑ in CRP (d28)
1-H10-C2	F	Chinese	~ 5	10	d0, 2, 3, 5, 7, 10, 14, 21, 28	Fever (d2, 3); anorexia (d1, 3); petechial rash (d3, 4, 7, 8); periorbital edema (d7-9); dark ocular discharge (d7). Subject survived to study endpoint (d28).	Lymphopenia (d2, 5); thrombocytopenia (d5, 7); monocytopenia (d5); granulopenia (d5-14); lymphocytosis (d10, 14); hypoalbuminemia (d5-14); hypoproteinemia (d7); > 2-fold ↑ in AST (d3-10); > 2-fold ↑ in ALP (d3-7)
2-H10-C1	F	Chinese	~ 4	10	d0, 2, 3, 5, 7, 10, 14, 21, 28	Anorexia (d1-7); petechial rash (d2-4). Subject survived to study endpoint (d28).	Lymphopenia (d2-5); thrombocytopenia (d3-7); monocytopenia (d2-7); granulopenia (d3-10); leukocytosis (d14); granulocytosis (d2-5, 14–28); > 2-fold ↑ in CRE (d5); hypoalbuminemia (d5-10); hyperamylasemia (d2); hypoamylasemia (d3-7, 21,28); > 3-fold ↑ in ALT (d2-14); > 3-fold ↑ in AST (d2-10); > 3-fold ↑ in CRP (d3)
2-H10-C2	M	Chinese	~ 4	10	d0, 2, 3, 5, 7, 10, 14, 21, 28	Anorexia (d1-3, 5–8); epistaxis (d1, 7). Subject survived to study endpoint (d28).	Lymphopenia (d2,5); thrombocytopenia (2–7); monocytopenia (d2); granulopenia (d5-10); monocytosis (d5, 14–28); hypoamylasemia; > 2-fold ↑ in ALT (d3,5,7); > 2-fold ↑ in AST (d2, 3, 5); > 7-fold ↑ in CRP (d2); > 2-fold ↑ in CRP (d3)
2-H10-M1	M	Mauritius	~ 4	10	d0, 2, 3, 5, 7, 10, 14, 21, 28	Anorexia (d2-10, 12); nasal exudate (d9). Subject survived to study endpoint (d28).	Lymphopenia (d2-5); thrombocytopenia (3–7); granulopenia (d3-7, 28); lymphocytosis (d10); monocytosis (d14); 2-fold ↑ in CRE (d14); > 2-fold ↑ in ALT (d2-5); ≥ 2-fold ↑ in AST (d2-5); > 18-fold ↑ in CRP (d2); > 3-fold ↑ in CRP (d3)
2-H10-M2	M	Mauritius	~ 4	10	d0, 2, 3, 5, 7, 10, 14, 21, 28	Anorexia (d1-13, 21–23, 25–28); nasal exudate (d9). Subject survived to study endpoint (d28).	Lymphopenia (d2-5); thrombocytopenia (2–7); monocytopenia (d2-5, 21); granulopenia (d5, 7, 28); lymphocytosis (d10, 14); monocytosis (d14, 28); granulocytosis (d2, 21); > 2-fold ↑ in ALT (d10, 14); > 3-fold ↑ in AST (d2-7); > 12-fold ↑ in CRP (d2); > 3-fold ↑ in CRP (d3)
2-H9-M1	F	Mauritius	~ 3	9	d0, 2, 3, 5, 7, 10, 14, 21, 28	Anorexia (d1-13, 15, 23, 25, 27, 28). Subject survived to study endpoint (d28).	Lymphopenia (d2-5); thrombocytopenia (5, 7); granulopenia (d2, 5, 7); lymphocytosis (d10); hyperamylasemia (d10); > 2-fold ↑ in AST (d3, 5, 7); > 4-fold ↑ in CRP (d2)
2-H9-C1	M	Chinese	~ 4	9	d0, 2, 3, 5, 7, 10, 14, 21, 28	Anorexia (d1-7, 9); petechial rash (d3, 4, 7). Subject survived to study endpoint (d28).	Lymphopenia (d2-5); thrombocytopenia (2–7); monocytopenia (d2); granulopenia (d3-10); monocytosis (d14,28); hyperamylasemia (d3); > 2-fold ↑ in AST (d3, 5, 7); > 18-fold ↑ in CRP (d2); > 2-fold ↑ in CRP (d21)
3-H7-C1	F	Chinese	~ 3	7	d0, 2, 3, 5, 7, 10, 14, 21, 28	Anorexia (d1-3, 11, 16). Subject survived to study endpoint (d28).	Lymphopenia (d2, 5); monocytopenia (d5, 7, 28); granulopenia (d3, 5); leukocytosis (d10); lymphocytosis (d10); granulocytosis (d10, 14); hypoalbuminemia (d28); > 4-fold ↑ in CRP (d2)
3-H7-C2	M	Chinese	~ 8	7	d0, 2, 3, 5, 7, 10, 14, 21, 28	Petechial rash (d7, 8). Subject survived to study endpoint (d28).	Lymphopenia (d2,5); monocytopenia (d2); granulopenia (d2, 3, 7); monocytosis (d3-10); > 2-fold ↑ in ALT (d2, 3, 7); 2-fold ↑ in AST (d2); 2-fold ↑ in CRP (d3)
4-H10-M1	M	Mauritius	~ 18	10	d0, 3, 5, 7, 14, 21	Anorexia (d1-8). Subject survived to study endpoint (d21).	Monocytopenia (d21); granulopenia (d5,7); lymphocytosis (d14); > 2-fold ↑ in ALT (d7); > 3-fold ↑ in AST (d5, 7); > 6-fold ↑ in CRP (d3)

Subject numbers are coded as [Experiment number]-[Virus and passage number]-[Animal origin and individual number]. Days after CCHFV Hoti challenge are in parentheses. Lymphopenia, granulopenia, monocytopenia, and thrombocytopenia are defined by a ≥35% drop in numbers of lymphocytes, granulocytes, monocytes, and platelets, respectively. Leukocytosis, lymphocytosis, monocytosis, and granulocytosis are defined by a two-fold or greater increase in numbers of white blood cells over baseline. Anorexia is defined as ≥25% decrease in baseline food intake. Fever is defined as a temperature more than 2.5°F over baseline, or at least 1.5°F over baseline and ≥ 103.5°F. Hypothermia is defined as a temperature ≤3.5°F below baseline. Hyperglycemia is defined as a two-fold or greater increase in levels of glucose. Hypoglycemia is defined by a≥25% decrease in levels of glucose. Hypoalbuminemia is defined by a ≥25% decrease in levels of albumin. Hypoproteinemia is defined by a ≥25% decrease in levels of total protein. Hyperamylasemia is defined by a ≥2-fold increase in levels of serum amylase. Hypoamylasemia is defined by a ≥25% decrease in levels of serum amylase. Hypocalcemia is defined by a ≥25% decrease in levels of serum calcium. (ALT) alanine aminotransferase, (AST) aspartate aminotransferase, (ALP) alkaline phosphatase, (CRE) Creatinine, (CRP) C-reactive protein, (Hct) hematocrit, (Hgb) hemoglobin

**Table 3:** Serum CCHFV neutralizing antibody titers of cynomolgus macaques.

		Serum CCHFV neutralizing antibody titer ^a^		
Challenge virus	Subject No.	Day 0^b^	Day 21^b^	Day 28^b^
Afghanistan	1-A5-C1	< 10	160	640
	1-A5-C2	< 10	640	1280
	4-A5-M1	< 10	80	N/A
	4-A5-M2	< 10	40	N/A
Hoti	1-H10-C1	< 10	320	1280
	1-H10-C2	< 10	1280	2560
	2-H10-C1	< 10	N/A	640
	2-H10-C2	< 10	N/A	1280
	2-H10-M1	< 10	N/A	640
	2-H10-M2	< 10	N/A	2560
	2-H9-M1	< 10	N/A	160
	2-H9-C1	< 10	N/A	1280
	3-H7-C1	< 10	N/A	160
	3-H7-C2	< 10	N/A	320
	4-H10-M1	< 10	320	N/A

^**a**^ Reciprocal serum dilutions at which 50% virus was neutralized.

^**b**^ Day after CCHFV challenge

N/A = not assessed

### NHP Experiments 2 and 3: Neither CCHFV Hoti passage number nor the origin of cynomolgus macaques influences clinical illness or pathogenesis

Given our unexpected result that CCHFV Hoti did not cause severe or lethal infection in either of the two Chinese origin cynomolgus macaques in the initial study, we performed a second study to further explore this model. The goal of this second study was to assess the effect of different experimental conditions from the previously published experiment [16], including the origin of the macaques employed as well as the passage history of the CCHFV Hoti challenge material. Our first study employed Chinese origin cynomolgus monkeys which are the most frequently used type of cynomolgus monkey used for infectious disease research [21], and are commonly used as uniformly lethal models of other important HF virus infections such as those caused by Ebola, Marburg, and Lassa viruses [22–26]. We hypothesized that a different origin of macaque, such as Mauritius origin cynomolgus monkeys, may be differentially susceptible to CCHFV given the more homologous genetic background of Mauritius-derived animals [27], which is thought to contribute to narrowed variability in development of immunity [28, 29]. Furthermore, marked variation in time to death to lethal infection has been observed in Ebola virus-infected Mauritius macaques compared with those of Chinese origin [30]. In addition, the CCHFV Hoti challenge material in our initial study was passaged one additional time than the virus stock previously reported to cause severe and lethal disease in macaques [16]. Therefore, we also utilized the identical stock of CCHFV Hoti virus from Haddock et al. [16] in this study. In brief, our second study employed six cynomolgus macaques: three of Chinese origin (subjects 2-H10-C1, 2-H10-C2, and 2-H9-C1) and three of Mauritius origin (subjects 2-H10-M1, 2-H10-M2, and 2-H9-M1). As we were limited in having only a very small volume of the lower passage (P9) CCHFV Hoti stock used in the previous reported work [16], we challenged one Chinese origin animal (subject 2-H9-C1) and one Mauritius origin animal (subject 2-H9-M1) with 2x10^5^ pfu of this material by the i.v. route. We then challenged two Chinese origin macaques (subjects 2-H10-C1 and 2-H10-C2) and two Mauritius origin macaques (subjects 2-H10-M1 and 2-H10-M2) with 2x10^5^ pfu by the i.v. route with the same P10 CCHFV Hoti stock used in our first study. Consistent with the results of our initial study, all six macaques developed a transient viremia between days 2 and 5 after CCHFV challenge which was accompanied by similar changes in clinical pathology, yet none of the animals developed severe disease (**Table 2**). As before, no significant lesions were observed upon postmortem examination or histopathological/immunohistochemical analysis of tissues from any of the animals, and all six macaques in this study developed neutralizing antibodies. (**Table 3**).

As our first two studies produced consistent findings and as it appeared that discrepancies between our findings and those of the previous report [16] were not likely due to the origin of the macaques, we further explored the potential influence of the passage history of the CCHFV Hoti stock in being responsible for the lesser severity of disease that we observed. To do so, we obtained the lowest passage of CCHFV Hoti stock that we could procure (P7) and used this stock to challenge two Chinese origin cynomolgus macaques (subjects 3-H7-C1 and 3-H7-C2) to 2x10^5^ pfu by the i.v. route. Consistent with the results of our first two studies, both animals developed a transient viremia; however, the duration of viremia was shortened to between days 2 and 3 after CCHFV challenge (**Fig 1**). Again, neither of the animals exhibited any gross pathological or histological lesions, and CCHFV antigen was absent in all tissues analyzed. As with the previous two studies, changes in hematology or serum biochemistry did not coincide with the development of severe disease (**Table 2**). Both animals survived to the study endpoint (day 28) and developed neutralizing antibodies to the challenge virus (**Table 3**).

### NHP Experiment 4: Age of cynomolgus macaques does not influence clinical outcome or pathogenesis after infection with CCHFV Hoti or Afghanistan isolates

In a final attempt to model lethal disease in humans and conceivably explain the discrepancy in our results versus those in Haddock et al. [16], we considered the age of the macaques used in these studies. In both the previous report and in our three studies, all of the animals ranged in age from 3 to 8 years. We procured three aged (> 18 years) Mauritius origin cynomolgus macaques. Two animals (subjects 4-A5-M1 and 4-A5-M2) were exposed to 2x10^5^ pfu of P5 CCHFV Afghanistan by the i.v. route and one animal (subject 4-H10-M1) was exposed to 2x10^5^ pfu of P10 CCHFV Hoti by the i.v route. Two animals (subjects 4-H10-M1and 4-A5-M1) developed viremia between days 2 and 7, with levels of infectious virus fluctuating between detectable and undetectable between days 3 and 7 in subject 41–873 (**Fig 1**). Subject 4-A5-M2 exhibited low level viremia on day 3 only. Likewise, despite some perturbations in hematology and serum biochemistry profiles, none of the animals developed severe disease (**Table 2**). All three animals had detectable neutralizing antibody titers by day 21 post-infection (**Table 3**).

### Analysis of serum cytokine/chemokines in cynomolgus monkeys following infection with CCHFV

**Fig 2:**
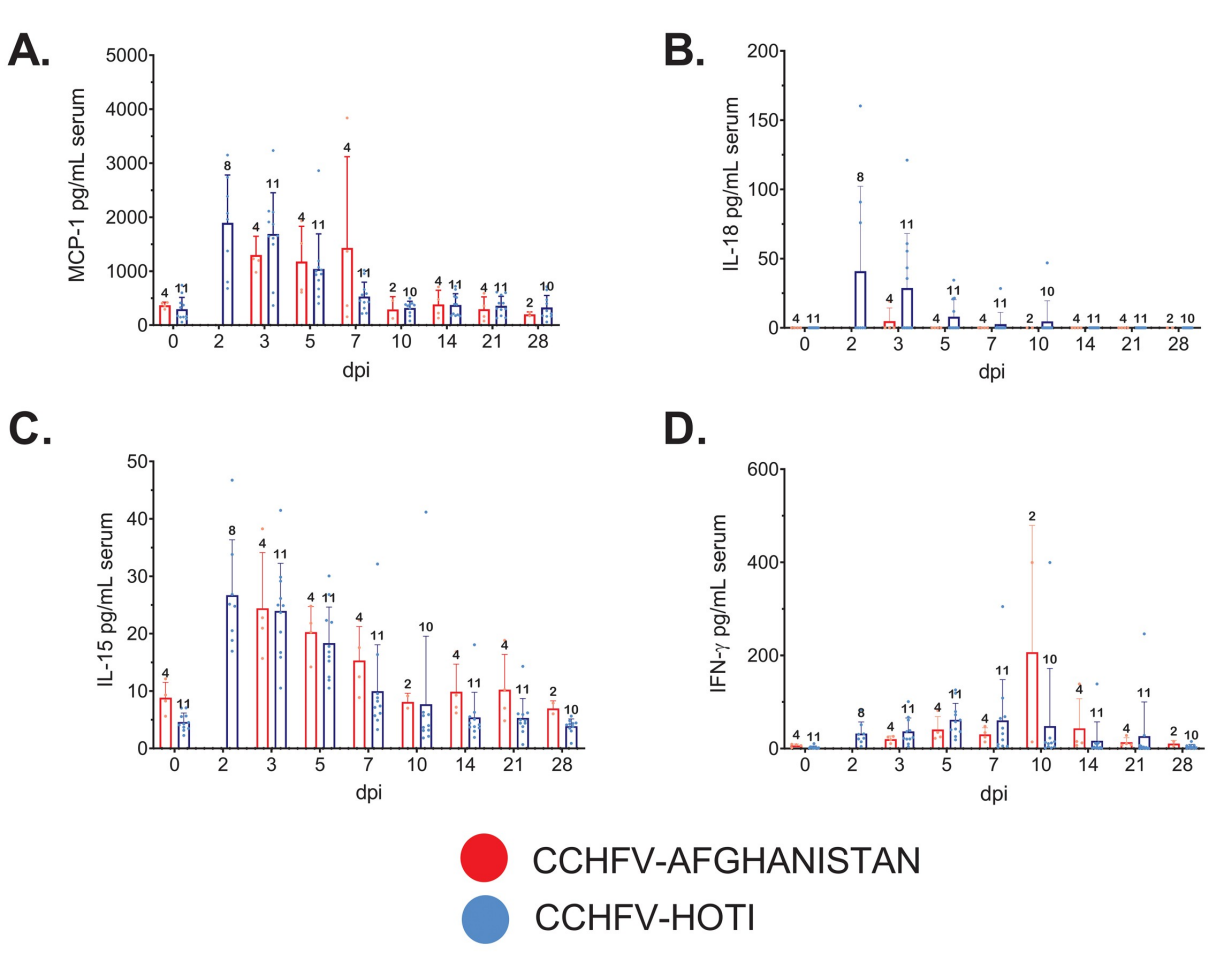
Serum cytokine/chemokine levels from CCHFV Afghanistan- and Hoti-infected cynomolgus macaques. Whole blood was collected from each animal at the indicated time points post infection and assayed for circulating serum cytokine and chemokines using a panel of 23 analytes (see Methods). Analytes depicted were chosen based on an obvious shift from baseline in most or all animals, their relevance in human infection, or for comparison against those reported in Haddock et al. [16]. Individual points (red or black circles) are the mean value of two technical replicates from the same sample, corresponding bars indicate the mean value for the group. Error bars indicate the upper SD. The number of individuals assayed at each time point (n) is indicated above error bar. **(A)** MCP-1; **(B)** IL-18; **(C)** IL-15; **(D)** IFN-γ. Note that due to differences in the sampling schedule between studies, data is not available for Afghanistan animals at the day 2 timepoint.

**Fig 3:**
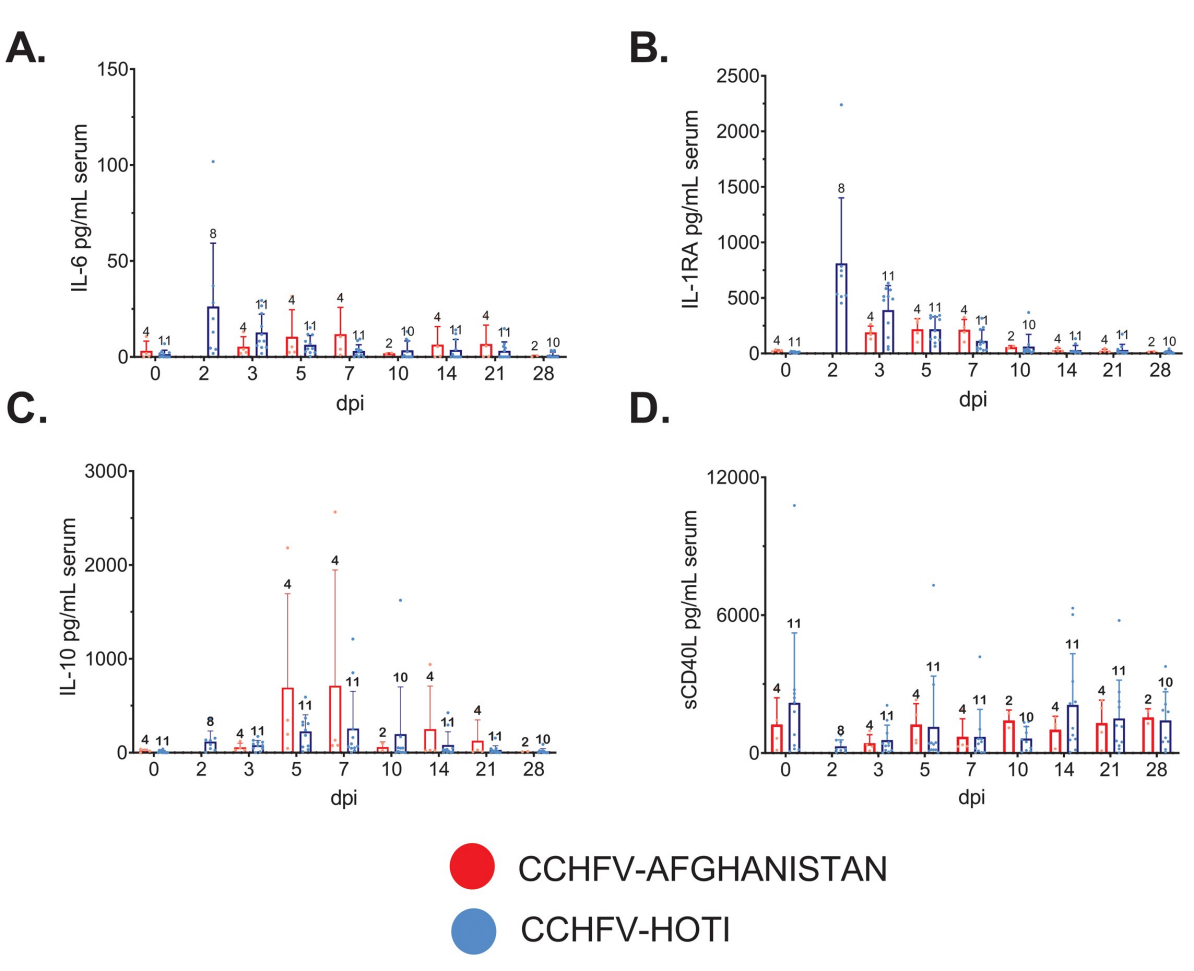
Serum cytokine/chemokine levels from CCHFV Afghanistan- and Hoti-infected cynomolgus macaques. Whole blood was collected from each animal at the indicated time points post infection and assayed for circulating serum cytokine and chemokines using a panel of 23 analytes (see Methods). Analytes depicted were chosen based on an obvious shift from baseline in most or all animals, their relevance in human infection, or for comparison against those reported in Haddock et al. (16). Individual points (red or black circles) are the mean value of two technical replicates from the same sample, corresponding bars indicate the mean value for the group. Error bars indicate the upper SD. The number of individuals assayed at each time point (n) is indicated above error bar. **(A)** IL-6; **(B)** IL-1RA; **(C)** IL-10; **(D)** sCD-40L. Note that due to differences in the sampling schedule between studies, data is not available for Afghanistan animals at the day 2 timepoint.

For all studies, a panel of serum cytokines/chemokines was assayed from blood collections on procedure days. Subjects were grouped by challenge virus strain for comparison. Similar to what was observed in the work by Haddock et al. [16], most analytes remained relatively static from baseline (day 0) through the study endpoint (day 21 or day 28), or showed marked variation between individual animals. However, several circulating analytes including IFN-gamma, IL-10, IL-6, IL-15, sCD40L, IL-1RA, MCP-1, and IL-18 were modestly changed over the course of infection but then returned to baseline shortly after the acute phase of disease (approximately day 5–7), indicative of a controlled response to infection (**Figs 2 and 3**). Early in infection, MCP-1 was markedly increased in parallel with induction of IL-18, a recognized inducer of myloid cell activation with a predilection for NK cells [31, 32]. In line with potential myloid activation (including natural killer and dendritic cells), early increases in IL-15 was followed by controlled induction and cessation of the potent antiviral IFN-γ, followed by reduction in viral genomes detected in blood [33, 34] (**Figs 1 and 2**). Early production of proinflammatory cytokine IL-6 occurred with a paralleled production of the anti-inflammatory IL-1RA from days 2–7, followed by increased levels of circulating IL-10, another potent anti-inflammatory cytokine. Interestingly, there was a marked depression in circulating sCD40L, a marker of platelet and T-cell activation that appeared to trend with the transient thrombocytopenia and lymphocytopenia observed early in infection (**Fig 3, Tables 1 and 2**).

**Fig 4:**
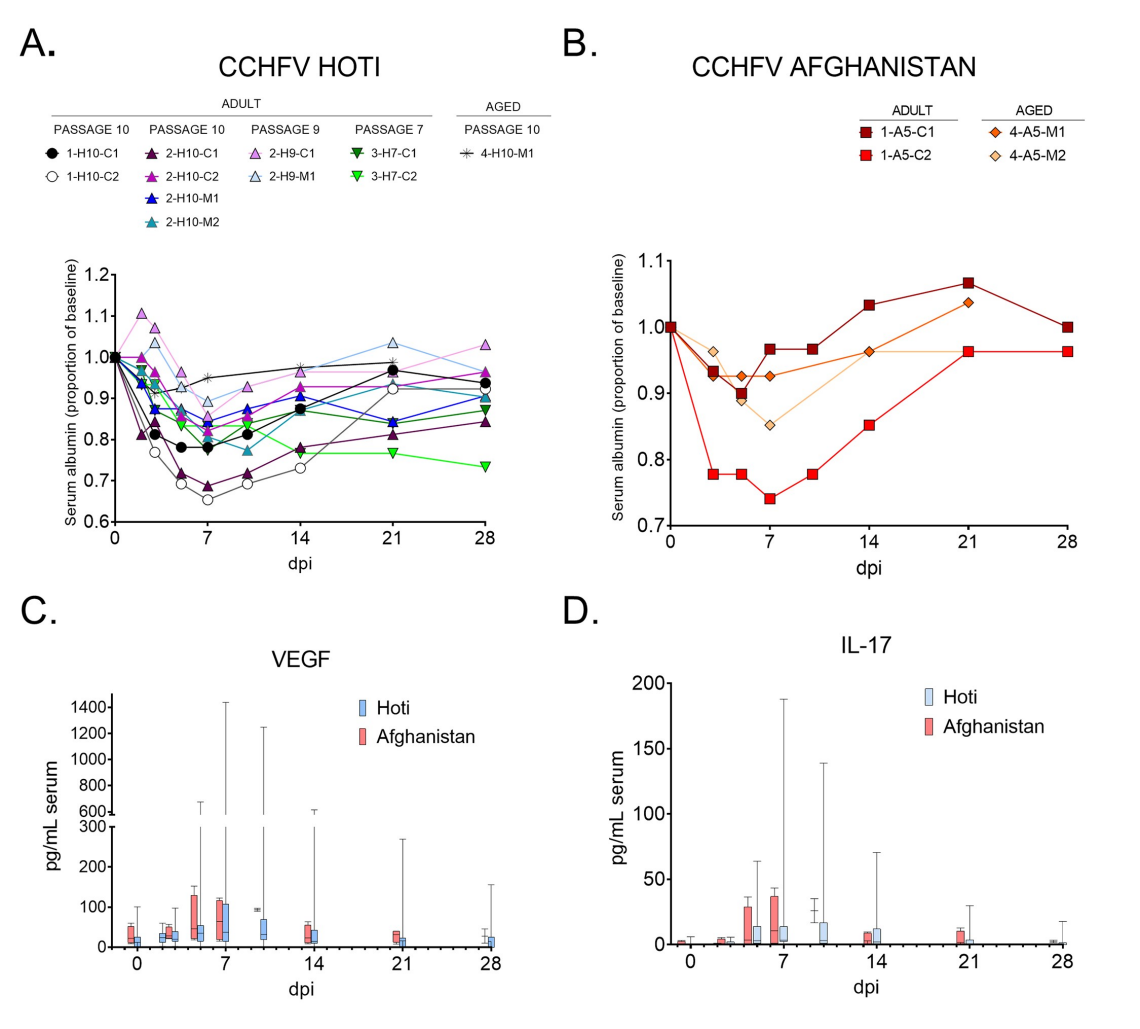
Transient vascular leakage in CCHFV infection of cynomolgus macaques. Proportions of circulating serum albumin (A and B) over the course of infection into recovery. Serum levels of VEGF (C) and IL-17 (D) are represented as box plots where boxes indicate range from 25th (bottom line) to 75th (top line) percentiles. The central line is the median value of all biological replicates and the whiskers represent the entire range of values (maximum to minimum). Days post infection = dpi.

Vascular leakage is a hallmark of many viral HFs including CCHFV [35–37]. Modest increases in VEGF and IL-17, which are associated with vascular function, coincided with decreases in circulating albumin and total protein in all animals to varying degrees of severity, with all of these markers returning to baseline by study endpoint (**Fig 4, S2 Fig**). Early studies on VEGF have determined it to be an incredibly potent agonist of vascular leakage, up to 50,000 times as potent as histamine [38]. IL-17, while not known to directly cause vascular leakage, is associated with enhanced nitric oxide production, a recognized potent vasomodulator [39, 40].

## Discussion

CCHF is the most widespread tick-borne viral disease of humans, causing high rates of morbidity and mortality. Prevention and containment of CCHF outbreaks presents logistical challenges for several reasons. Tick vectors are prevalent and widespread, and CCHFV infection of domestic animals goes largely unnoticed. The problem is further amplified by the lack of an effective and available vaccine. In order to advance the development of candidate vaccines and treatments for emerging viral pathogens like CCHFV, animal models that mimic human disease are needed. Until recently, there were no animal models for CCHF. Over the last decade several immunocompromised mouse models of CCHF have been developed [8, 9]. While these models may have some utility for triaging antivirals and have been used to assess CCHFV vaccines [11, 19, 41–47], they are not ideal, as these mice do not have intact immune systems and thus may not satisfy regulatory requirements for vaccine licensure under the US FDA Animal Rule [48].

The recent report of an immunocompetent NHP model of CCHF was a major advance in being able to assess the efficacy of candidate countermeasures [16]. In order to confirm this CCHF model and further develop additional CCHF NHP models we exposed eleven cynomolgus macaques to CCHFV Hoti and four cynomolgus macaques to CCHFV Afghanistan. Surprisingly, under near identical test conditions we were unable to reproduce the severe disease and lethality seen in the previous study. Despite developing transient viremia and seroconversion, indications that infection with CCHFV did occur, all animals in our study only developed mild clinical disease when challenged with either the Hoti or Afghanistan strains of CCHFV. However, it should be noted that in the Haddock et al. study [16], the authors did observe asymptomatic infection in some animals challenged with CCHFV Hoti by a different route (sub-cutaneous). In addition, a recent report by Smith et al. also assessed the pathogenic potential of the Hoti and Afghanistan strains in cynomolgus macaques, and similarly observed a lack of severe or lethal disease [49]. However, the interpretation of their findings regarding the host response to infection is complicated by the fact that a substantial portion of the animals in their study were found to be latently infected with *Mycobacterium tuberculosis*. While the results of this previous study may have utility in assessing co-infection, a direct comparison to the study performed by Haddock et al. [16] is challenging given this confounding factor. In an attempt to understand the discrepancy between our study and Haddock et al. [16], we employed a number of different experimental conditions including using CCHFV Hoti stocks with different passage histories and employing cynomologus macaques of different origin as well as using aged (>18 years) animals. None of these altered test conditions affected the clinical outcome observed in the initial experiment. While we cannot exclude other unknown variables for the different results between our studies and those in Haddock et al. [16], the most likely explanation is the outbred nature of cynomologus macaques, where small genetic differences from animal to animal may impact the outcome, as has been postulated for variable challenge outcomes with other viruses, such as SARS coronavirus, where a similar inconsistency in disease modeling between studies in macaques is apparent [50]. CCHFV infection of humans results in an average case fatality rate of 30% with fatality rates as low as 5% for outbreaks in some regions. Therefore, while it would be advantageous for screening of vaccines and therapies to have a uniformly lethal NHP model, it may not be easily achievable. There are other examples of viral infection of NHPs that produce a broad spectrum of disease from mild illness to severe disease and fatal infection such as with Lassa virus infection of rhesus and cynomolgus macaques [51]. The use of small numbers of animals in BSL-4 studies common to most BSL-4 facilities for logistical, financial, and ethical considerations may also contribute to disparate results among studies. This may be particularly more noticeable in animal models where disease manifestations vary and there is not uniform severe illness or lethality.

There is also great utility in models of varied lethality in that it affords the opportunity to study disease processes associated with early signals of disease as well as markers of survival and or persistence. For example, cytokines-chemokines and comparisons with viral clearance and comparison with human disease. Indeed, aberrations in levels of MCP-1, IL-1RA, IL-6, IFN-γ, IL-10, VEGF-A, and sCD-40L have all been detected in humans [52–54]. While only a small number of clinical studies have been performed, taking information from animal models has power in identifying new biomarkers which may play important roles in human disease severity and recovery from infection. For instance, elevated levels of circulating nitric oxide intermediates have been detected in human CCHF disease [55] which raises potential connection with elevated IL-17 levels in this study observed in this study.

In summary, the CCHFV Hoti and CCHFV Afghanistan macaque models should prove valuable for assessing promising interventions. While we did not observe severe or lethal CCHF disease, other NHP models, such as influenza [56], SARS [50], and Zika [57] also use biomarkers of infection, such as viremia and changes in clinical pathology parameters, to assess efficacy of medical countermeasures.

## Supporting information

S1 Fig Hematoxylin/eosin and immunohistochemical staining of tissues from CCHFV-challenged cynomolgus macaques and comparison to mouse controls. Representative H&E-stained tissue specimens **(A,C,E,G,I,K,M,O,Q,S)** and IHC antibody labeled tissue specimens (**B,D,F,H,J,L,N,P,R,T**). For IHC images, CCHFV antigen labeling (NP protein), if present, is shown in brown. Panels **A,B,E,F,I,J,M,N** are from subject 1-A5-C2, challenged with the Afghanistan isolate, and are representative of what was observed in all subjects challenged with this isolate. Panels **C,D,G,H,K,L,O,P** are from subject 1-H10-C2, challenged with the Hoti isolate, and are representative of what was observed in all subjects challenged with this isolate. Panels **Q** and **R** are of the liver from a historical control mouse that succumbed to infection by the Afghanistan isolate. Panels **S** and **T** are of the liver from a historical control mouse that succumbed to infection by the Hoti isolate. All images were captured at 20X magnification.
(TIF)

S2 Fig Circulating total protein content in from CCHFV Afghanistan- and Hoti-infected cynomolgus macaques. Proportions of circulating total protein from CCHFV-Hoti (A) and Afghanistan (B) challenged animals over the course of infection into recovery.
(TIF)
