# Supplementary figures and images for: Crimean-Congo hemorrhagic fever virus strains Hoti and Afghanistan cause viremia and mild clinical disease in cynomolgus monkeys
Source: PLoS Negl Trop Dis. 2020 Aug 13;14(8):e0008637. doi: 10.1371/journal.pntd.0008637 (PMC7447009; doi:10.1371/journal.pntd.0008637)

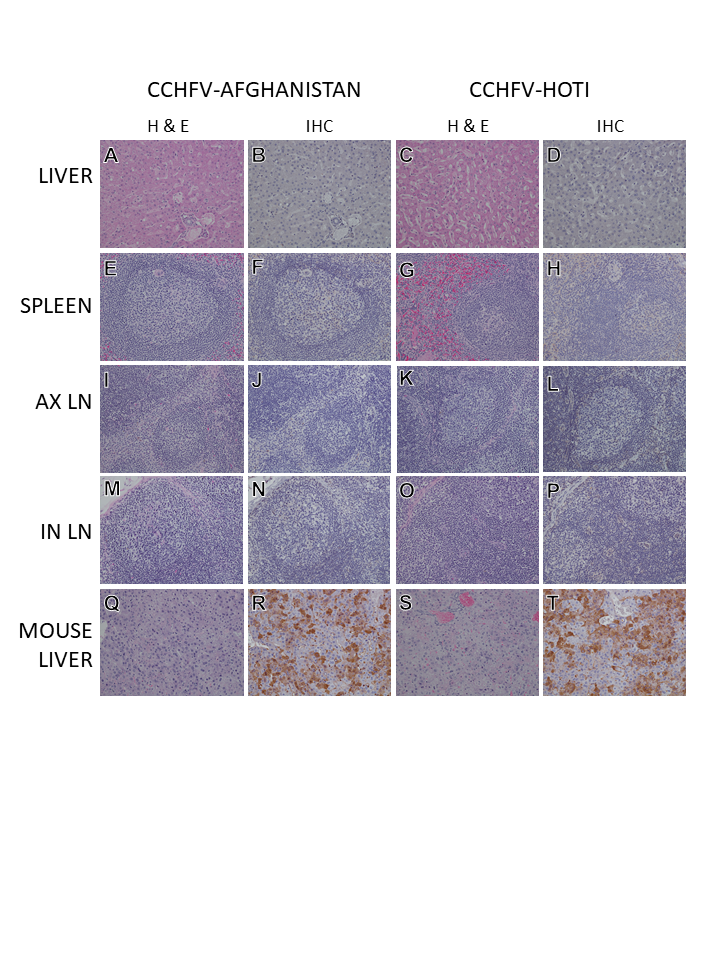

Supplement: S1 Fig — Representative H&E-stained tissue specimens (A,C,E,G,I,K,M,O,Q,S) and IHC antibody labeled tissue specimens (B,D,F,H,J,L,N,P,R,T). For IHC images, CCHFV antigen labeling (NP protein), if present, is shown in brown. Panels A,B,E,F,I,J,M,N are from subject 1-A5-C2, challenged with the Afghanistan isolate, and are representative of what was observed in all subjects challenged with this isolate. Panels C,D,G,H,K,L,O,P are from subject 1-H10-C2, challenged with the Hoti isolate, and are representative of what was observed in all subjects challenged with this isolate. Panels Q and R are of the liver from a historical control mouse that succumbed to infection by the Afghanistan isolate. Panels S and T are of the liver from a historical control mouse that succumbed to infection by the Hoti isolate. All images were captured at 20X magnification. (TIF) [file pntd.0008637.s001.tif]
